# Resident memory T cells, critical components in tumor immunology

**DOI:** 10.1186/s40425-018-0399-6

**Published:** 2018-09-04

**Authors:** Fathia Mami-Chouaib, Charlotte Blanc, Stéphanie Corgnac, Sophie Hans, Ines Malenica, Clémence Granier, Isabelle Tihy, Eric Tartour

**Affiliations:** 1 INSERM UMR 1186, Integrative Tumor Immunology and Genetic Oncology, Gustave Roussy, EPHE, PSL, Faculté de Médecine, University Paris-Sud, Université Paris-Saclay, 39, rue Camille Desmoulins, F-94805 Villejuif, France; 2 INSERM U970, Universite Paris Descartes, Paris, France; 3 Hôpital européen Georges Pompidou. Service d’Immunologie biologique, 20, Rue Leblanc, 75015 Paris, France; 4 Equipe labellisée Ligue contre le Cancer, Paris, France

**Keywords:** Resident memory T cells, Immune checkpoint receptors, Immunotherapy, CD103 integrin, Immunosurveillance

## Abstract

CD8^+^ T lymphocytes are the major anti-tumor effector cells. Most cancer immunotherapeutic approaches seek to amplify cytotoxic T lymphocytes (CTL) specific to malignant cells. A recently identified subpopulation of memory CD8^+^ T cells, named tissue-resident memory T (T_RM_) cells, persists in peripheral tissues and does not recirculate. This T-cell subset is considered an independent memory T-cell lineage with a specific profile of transcription factors, including Runx3^+^, Notch^+^, Hobit^+^, Blimp1^+^, BATF^+^, AHR^+^, EOMES^neg^ and Tbet^low^. It is defined by expression of CD103 (α_E_(CD103)β_7_) and CD49a (VLA-1 or α_1_β_1_) integrins and C-type lectin CD69, which are most likely involved in retention of T_RM_ cells in non-lymphoid tissues, including solid tumors. CD103 binds to the epithelial cell marker E-cadherin, thereby favoring the location and retention of T_RM_ in epithelial tumor regions in close contact with malignant cells. The CD103-E-cadherin interaction is required for polarized exocytosis of lytic granules, in particular, when ICAM-1 expression on cancer cells is missing, leading to target cell death. T_RM_ cells also express high levels of granzyme B, IFNγ and TNFα, supporting their cytotoxic features. Moreover, the local route of immunization targeting tissue dendritic cells (DC), and the presence of environmental factors (i.e. TGF-β, IL-33 and IL-15), promote differentiation of this T-cell subtype. In both spontaneous tumor models and engrafted tumors, natural T_RM_ cells or cancer-vaccine-induced T_RM_ directly control tumor growth. In line with these results, T_RM_ infiltration into various human cancers, including lung cancer, are correlated with better clinical outcome in both univariate and multivariate analyses independently of CD8^+^ T cells. T_RM_ cells also predominantly express checkpoint receptors such as PD-1, CTLA-4 and Tim-3. Blockade of PD-1 with neutralizing antibodies on T_RM_ cells isolated from human lung cancer promotes cytolytic activity toward autologous tumor cells*.* Thus, T_RM_ cells appear to represent important components in tumor immune surveillance. Their induction by cancer vaccines or other immunotherapeutic approaches may be critical for the success of these treatments. Several arguments, such as their close contact with tumor cells, dominant expression of checkpoint receptors and their recognition of cancer cells, strongly suggest that they may be involved in the success of immune checkpoint inhibitors in various cancers.

## Background

Conventional memory T cells classically include central memory T (T_CM_) cells, residing in lymphoid organs and reactivated during secondary infection, and effector memory T (T_EM_) cells, circulating through various tissues and endowed with cytotoxic properties. A population of memory T cells, named tissue resident memory T (T_RM_) cells, has been recently identified. These memory T cells persist in tissues and do not recirculate [1–6]. A seminal work from Klonowski et al. showed limited mixing of blood CD8^+^ T lymphocytes with intraepithelial lymphocytes (IEL) from the lamina propria and the brain between mice joined by parabiosis [7]. Then, D Masopust in the group of R Ahmed demonstrated the residency of intestinal memory CD8^+^ T cells after transplantation of gut harboring memory CD8^+^ T cells into naive mice [8] and Steinert et al., by parabiosis experiments [9]. These cells appear to be generated from a subpopulation of T_EM_ cells patrolling and surveying the tissue. T_CM_ cells have also the ability to differentiate into T_RM_ cells after reactivation and acquisition of CD69 molecule leading to retention in the tissue [10]. A body of evidence has shown that T_RM_ cells represent an independent memory T-cell lineage with a specific differentiation pathway. Unexpectedly, transcription factors that have usually been associated with long-lived memory cells, such as eomesodermin (EOMES) and transcription factor 1 (TCF1, also known as HNF1α) [11, 12], are not expressed in T_RM_ cells. In mice, but not in humans, the combined loss of Hobit and Blimp-1 transcription factors strongly compromised development of T_RM_ [13, 14]. Recent studies identified Notch and Runx3 transcription factors as master regulators in induction and maintenance of human CD8^+^ T_RM_ cells [14, 15]. Accessible chromatin regions were identified in IEL mature TRM cells near genes characteristically expressed in mature TRM cells (such as *Cd69* and *Nr4a1*), whereas genes that promote T-cell recirculation (such as *Klf2* and *S1pr1*) exhibited loss of accessible regions [14, 15]. The latter study also demonstrated that CD8^+^ T cells localized in non-lymphoid tissues have a global chromatin landscape that differed from that of lymphoid effector CD8^+^ T cells, reinforcing the unique features of the T_RM_ cell subset [15]. The cytokines interleukin-33 (IL-33) and tumor necrosis factor (TNF), in combination with TGF-β, can induce a T_RM_ cell-like phenotype [16], as well as downregulation of *KLF2* expression in CD8^+^ T cells [17]. KLF2 promotes expression of genes such as *sphingosine-1-phosphate receptor 1* (*S1PR1*), favoring the egress of T cells from tissues [17].

T_RM_ cells play an essential role in protecting human epithelial tissues against infectious and inflammatory diseases. They are highly activated T lymphocytes that reside within a variety of peripheral tissues, including intestine [8, 18], brain [19], skin [3, 20] and lung [21], and they provide rapid and effective responses to viral reinfections [22]. This T-cell subset is defined by expression of CD103 (α_E_(CD103)β_7_) and CD49a (VLA-1 or α_1_β_1_) integrins and the C-type lectin CD69. This phenotype may explain the retention of T_RM_ cells in tissue. Indeed, a role for CD103 in T-cell homing into epithelia has been previously suggested [23–25]. Along the same lines, an enhanced CD103^+^ TIL subset correlated with increased intraepithelial lymphocyte infiltration [26, 27], supporting the hypothesis that CD103 promotes recruitment of T_RM_ cells within epithelial tumor islets. The intra-epithelial location of CD103^+^CD8^+^ T cells was also observed in colorectal and bladder cancers, and was associated with expression of E-cadherin on tumor cells [28, 29]. Consistently, studies performed with viable human tumor slices [30] and autologous tumor antigen-specific CTL clones showed that CD103 contributes to T-cell recruitment within epithelial tumor regions and enhances intratumoral T-cell early signaling [31]. Indeed, recruitment of CD8^+^ T lymphocytes within epithelial tumor islets was inhibited by anti-CD103 neutralizing monoclonal antibodies (mAb), while TGF-β enhanced CD103-dependent T-cell movement toward epithelial tumor regions [31]. In this context, studies from one of our groups showed that CD103 mediates arrest of T lymphocytes under flow by interacting with E-cadherin on epithelial tumors [32]. Moreover, interaction of CD103 with E-cadherin promotes CCR5 recruitment at the immune synapse formed between T_RM_ cells and tumor target cells, leading to inhibition of T-cell sensitivity to the CCL5 chemotactic gradient [33].

CD49a is not required for initial recruitment of effector CD8^+^ T cells, but is critically important in their retention during the memory phase [34, 35]. We and other groups have shown that the number of T_RM_ cells is reduced in peripheral tissues after injection of anti-VLA-1 blocking antibodies at the memory phase of the immune response [34], as well as in tumors [36]. The CD69 molecule downregulates expression of S1PR1, which favors the exit of T cells from tissues [37]. Intra-tumoral CD8^+^ T_RM_ cells express high levels CD69 and concomitantly low levels of S1PR1, which prevent their recirculation in the bloodstream and their migration into lymphoid organs [17, 38]. Moreover, parabiosis experiments demonstrated that T_RM_ cells induced after therapeutic cancer vaccination were unable to migrate toward the non-immunized parabiont, supporting their tissue residency features [39]. It is noteworthy that some CD8^+^ T_RM_ cells lack CD103, and that this integrin is not an absolute marker for residency of CD4^+^ T_RM_ [40, 41]. For example, CD4^+^ memory T cells in human dermis lack CD103 expression, whereas those in the epidermis are CD103^+^ [42]. Notably, the presence of T_RM_ cells in human epithelial tumors and the role of this T-cell subset in anti-tumor immunity have thus far not been systematically addressed. Accumulating evidence indicates that T_RM_ cells also frequently reside in human tumors, especially of epithelial origin, and play an essential role in tumor-specific T-cell responses (for a review see [43]).

## Phenotypic features of T_RM_ cells in cancer

**Fig. 1:**
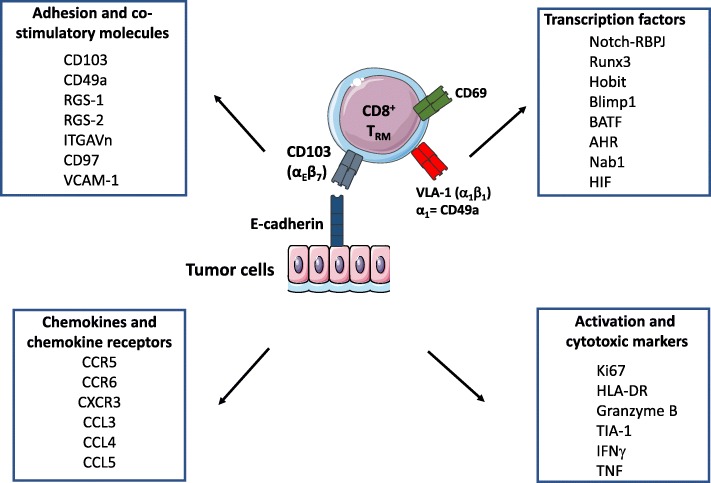
Core signature of resident memory T cells. Results from transcriptomic and cytometry analyses define some core markers belonging to family of molecules (adhesion/costimulatory molecules, chemokines and chemokine receptors, activation and cytotoxic markers, and transcription factors, etc.). However, the phenotype of T_RM_ cells may vary depending on their location

Previous studies from one of our groups revealed that human lung tumor-infiltrating lymphocytes (TIL) include a homogeneous CD8^+^ T-cell population defined by expression of CD103 and CD69 [27]. T_RM_ cells do not express CCR7, CD62L or S1PR1 [14, 27, 38], which are required for tissue exit (Fig. 1). This CD103^+^CD8^+^ T-cell subset displays a unique transcriptomic signature characteristic of T_RM_ cells, with upregulation of retention and adhesion-molecule-encoding genes such as *RGS1, RGS2*, *ITGA1*, *ITGAV* and *VCAM1* [14, 27]. This TIL subpopulation also expresses a broad range of chemokine receptors, including CXCR3, CCR5 and CCR6, and was able to produce chemokines such as CCL3, CCL4, CCL5, and inflammatory cytokines such as IFNγ and TNF. T_RM_ cells also express the pro-survival family member Bcl-2, as well as anti-apoptotic factors, including PHLDA1 and BIRC3, which may explain their long survival in tissues [14, 27].

Furthermore, there is a cluster of transcription factors associated with T_RM_. These factors include activator protein AP-1, Notch1-RBPJ (RBPJ is also known as CSL) and NF-κB transcription factor complexes, as well as BATF (basic leucine zipper transcription factor) and AHR (aryl hydrocarbon receptor), which regulate expression of homing receptors and maintenance of mouse T_RM_ cells, respectively [38, 44, 45]. BATF has also been shown to regulate the metabolism and survival of CD8^+^ T cells [46, 47]. Residual Tbet expression in T_RM_ promotes expression of IL-15R, which is critical for T_RM_ survival and functions [48]. However, overexpression of Tbet transcription factor inhibits the generation of T_RM_ cells. Remarkably_,_ human infant T cells exhibit increased expression of Tbet compared with adult T cells, leading to a preferential generation of effector T cells over T_RM_ cells [49, 50]. This data may explain that infants suffer disproportionately from respiratory infections.

NAB1 is a transcription factor overexpressed in T_RM_ cells, the mouse homolog of which (NAB2) is induced in CD8^+^ T cells that have received help from CD4^+^ T cells, and is needed to prevent activation-induced cell death (AICD) of those ‘helped’ CD8^+^ T cells [51]. T_RM_ also exhibited a glucose-deprivation signature, consistent with a lower glucose concentration in airway fluid than in blood. In lung cancer, T_RM_ cells had elevated expression of genes related to hypoxia, such as *HIF1A* (which encodes HIF-1α) and *EPAS1* (which encodes HIF-2α) [14].

## Mechanisms of action of T_RM_ cells

### Role of CD103 integrin

**Fig. 2:**
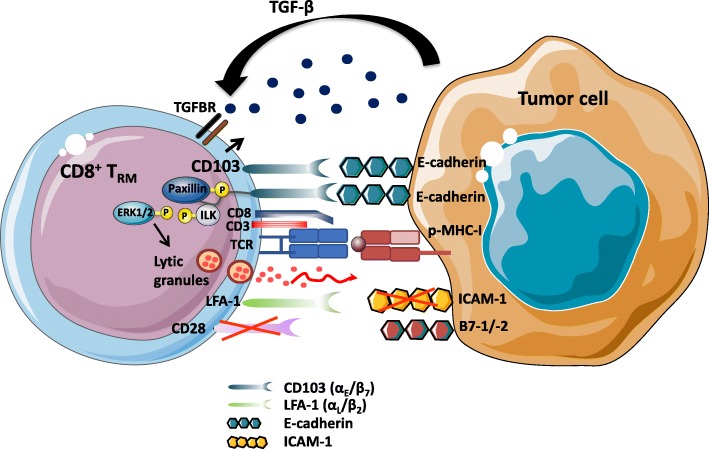
Role of CD103 integrin in anti-tumor T_RM_ functional activities. Engagement of TCR with specific peptide-MHC-I (p-MHC-I) complexes in the presence of TGF-β, abundant within the tumor microenvironment, induces expression of CD103 on the CD8^+^ T-lymphocyte surface. Phosphorylation of integrin-linked kinase (ILK) by TGFBR1, and its subsequent binding to the CD103 intracellular domain promotes inside-out signaling resulting in an increase in the affinity of the integrin for its ligand E-cadherin on tumor cells. Activated CD103 is recruited at the immune synapse formed between stimulated T_RM_ cells and epithelial target cells; its interaction with E-cadherin triggers phosphorylation of extracellular signal-regulated kinases 1 and 2 (ERK1/2) and the paxillin adaptor protein. Binding of phosphorylated (p)-paxillin to the α_E_ (CD103) subunit tail triggers an outside-in signal that promotes CD8^+^ T-cell effector functions such as cytokine production and polarized release of cytotoxic granules, leading to TCR-mediated target cell death. Intra-tumoral T_RM_ cells express very low levels of CD28 co-stimulatory receptor. Moreover, expression of LFA-1 on TIL is downregulated by TGF-β. Finally, cancer cells often downregulate expression of the LFA-1 ligand ICAM-1 to escape from immune effector cells

CD103 integrin is a heterodimeric transmembrane receptor formed by α_E_ (CD103) and β_7_ subunits, with the epithelial cell marker E-cadherin as a unique known ligand [52]. This integrin is expressed on T cells residing in tissue microenvironments, where TGF-β is abundant, such as mucosal CD8^+^ T lymphocytes and, mainly, IEL [53], but it is also expressed on CD4^+^ and CD8^+^ regulatory T (Treg) cells [54, 55] and on a large proportion of CD8^+^ effector T cells infiltrating epithelial tumors, including bladder [56], pancreatic [57], colorectal [28], ovarian [26] and lung cancers [27, 38, 58, 59]. It is induced on tumor-specific CD8^+^ T cells by concomitant signals from the TGF-β receptor (TGFBR) and the T-cell receptor (TCR) triggered by TGF-β and major histocompatibility complex class I (MHC-I)/tumor peptide complexes, respectively (Fig. 2) [33, 58, 60]. In this regard, adoptive transfer of tumor-specific CD8^+^CD103^+-^T cells in the cognate tumor engrafted in nonobese diabetic/severe combined immunodeficient (NOD/SCID) mice and subsequent coengagement of TCR and TGFBR trigger CD103 expression on T-cell surface associated with acquisition of a strong cytotoxic capacity toward autologous tumor cells. In contrast, adoptive transfer of these CD8^+^CD103^−^ T cells in allogeneic tumor does not result in expression of CD103 [33, 58]. Along the same line, CD103 is induced on tumor-specific T cells upon engagement of TCR with anti-CD3 mAb and TGF-β treatment [58, 60, 61]. In contrast, TGF-β alone had only a slight effect on CD103 expression and anti-CD3 mAb alone had no effect [58]. It is well known that only 1 to 3% of human circulating T cells expressed CD103, which implies that tumor-specific T cells need to encounter the cognate antigen within a TGF-β-rich tumor microenvironment to induce expression of the integrin and to become T_RM_.

CD103 appears to be a key molecule in T-cell activation and functions within the tumor microenvironment. Accordingly, a correlation between the expression level of CD103 on tumor-specific T-cell clones, stimulated in vitro with IL-2 and irradiated autologous tumor cells, and their capacity to kill autologous E-cadherin^+^ tumor cells was observed [58]. Indeed, CD103 is recruited at the immune synapse formed between CTL and epithelial tumor cells, and its interaction with E-cadherin is required for polarized exocytosis of lytic granules, leading to target cell lysis (Fig. 2). Moreover, killing of target cells was abrogated by anti-CD103 neutralizing mAb and siRNA targeting E-cadherin, pointing to a major role for the CD103-E-cadherin interaction in the anti-tumor CTL response. CD103 also helps in secretion of cytokines by tumor-specific CTL by interacting with E-cadherin on target cells [32]. This integrin is essential in controlling CD8^+^ TIL activities, not only by promoting effector T-cell adhesion to tumor cells, but also by triggering intracellular signaling events that co-stimulate TCR signals [59]. Indeed, binding of CD103 on freshly isolated tumor-infiltrating CD8^+^ T_RM_ cells to immobilized recombinant E-cadherin-Fc is sufficient to induce re-localization of cytolytic granules at the contact area, while degranulation requires TCR co-engagement. Moreover, this minimal triggering of CD103 promotes phosphorylation of ERK1/2 and phospholipase C (PLC)γ1, resulting in granule polarization and hence TCR-mediated cytotoxicity.

Activation of integrins on T cells is regulated by “inside-out” signaling, initiated by TCR and chemokine receptor stimulation, inducing integrin-extended conformation and clustering, thereby increasing their affinity for their ligands [62]. Remarkably, data from one of our groups indicated that TGF-β not only participates in CD103 induction on TCR-engaged antigen-specific CD8^+^ T cells [33, 58, 60], but is also directly involved in CD103 activation (Fig. 2). Indeed, TGF-β triggers TGFΒR1-mediated phosphorylation of ILK and its subsequent binding to the integrin intracellular domain, resulting in AKT phosphorylation and thereby initiating inside-out signaling that leads to increased CD103 affinity for its ligand E-cadherin [31]. Firm adhesion of CD103 to E-cadherin triggers phosphorylation of Pyk2 protein tyrosine kinase and the paxillin adaptor protein, and subsequent binding of phosphorylated-paxillin to the CD103 cytoplasmic domain, initiating outside-in signaling that promotes CD8^+^ T_RM_ migratory behavior and effector functions [63].

### Tumor T_RM_ cells are endowed with cytotoxicity potential

Lung T_RM_ cells display high expression levels of mRNA encoding effector molecules, such as granzyme B, IFN-γ and TNF, without the need for ex vivo stimulation [14]. In human lung tumors, CD8^+^CD103^+^ T_RM_ cells were also found to express mRNA encoding molecules associated with cytotoxic activities of killer cells, such as *IFNG*, *GZMA*, *GZMB* and *RAB27A* [27, 38, 64]. Expression by CD8^+^CD103^+^ TIL of granzyme B, perforin and the degranulation marker LAMP-1 (CD107a) was confirmed at the protein level, further supporting their cytotoxic potential [27, 38, 64]. Moreover, in ovarian and lung cancer, T_RM_ cells express the activation marker HLA-DR and the proliferation marker Ki67 [38, 64]. These cells may be functionally exhausted within the tumor microenvironment by the induction of T-cell inhibitory receptors including PD-1 and Tim-3 [27].

## Priming of T_RM_ cells in normal and tumoral tissues

T_RM_ cells are part of the adaptive immune system. Thus, their induction requires previous contact with antigenic peptide-MHC-I (pMHC-I) complexes following presentation of the antigen by antigen-presenting cells (APC). Notably, only particular subpopulations of DC (Batf3-dependent DC in mice, CD1c^+^ TGF-β-producing DC in human) have the ability to generate T_RM_ cells [10, 65]. The specificity of T_RM_ cells compared to other T cells resides in the differentiation cues after initial activation, which leads to expression of markers involved in T-cell residency and persistence in the tissue, such as CD103, CD49a and α_4_β_7_ [66]. Indeed, it has been shown by several groups that neutralization of T_RM_ cell markers by blocking antibodies toward CD49a or CD103, or the absence of α_4_β_7_ integrin in α_4_β_7_-deficient cells, hampers the presence and persistence of T_RM_ in tissues [36, 37, 67], suggesting a crucial role for these molecules in the development of T_RM_.

Cytokines present in tissues also contribute to induction of T_RM_ residency markers. As mentioned above, TGF-β, a cytokine produced by immune and epithelial cells, drives CD103 expression. In this context, inhibition of TGF-β by neutralizing antibodies or inactivation of its receptor on CD8^+^ T cells results in a decrease in CD8^+^CD103^+^ T-cell number in the specific tissue [39]. Depending on their location in the skin, salivary gland or kidney, T_RM_ cells require IL-15 for their persistence, likely due to the role of this interleukin in upregulation of CD103; but this does not seem to be the case in genital or small intestine tissues [37, 48, 68]. Furthermore, other environmental cues driven by retinoic acid or the pro-inflammatory cytokines IL-2, IL-12, IL-18 and IL-33, might also be essential, parallel to TCR activation, for development of the T_RM_ phenotype [17]. Various cells can secrete these molecules, including APC, stroma cells and epithelial cells. Consequently, the phenotype of T_RM_ depends on the cytokine profile present in each histological zone and, concomitantly, on secreting cells that infiltrate tissues [69], which may explain T_RM_ phenotypic diversity in the organism. Despite enhancing the formation of memory CD8^+^ T cells in secondary lymphoid tissues, rapamycin, an inhibitor of the mTor pathway, blocks the formation of resident memory CD8^+^ T cells in intestinal and vaginal mucosa. The ability of rapamycin to inhibit the formation of functional resident CD8^+^ T cells in mucosal tissues protected mice from a CD8^+^ T-cell-mediated lethal intestinal autoimmunity [70].

The role of cognate antigen in the priming of T_RM_ cells is a matter of debate, as in the lung and brain, this antigenic contact is mandatory [19, 71]. However, topical application of the skin irritant DNFB (2,4-dinitrofluorobenzene) or a local application of cytokines in the genital tract after systemic priming were sufficient for local generation and/or recruitment of T cells with a T_RM_ phenotype [72, 73]. In parallel with the molecular description, it has been shown that some vaccination strategies preferentially lead to induction of T_RM_ cells. Indeed, mucosal, but not systemic, routes (intramuscular), generate a potent local T-cell response with a T_RM_ phenotype, in parallel with a systemic response. For example, multiple studies, especially in an infectious context, have shown that tissue-specific vaccination is more effective at generating local immunity and T_RM_ cells at barrier sites because it favors homing of immune cells to local sites [74]. In the same manner, heterologous prime-boost strategy with a cervico-vaginal boost enhances the establishment of specific CD8^+^ T cells expressing α_4_β_7_ integrin in the genital tract compared to an intramuscular boost [67]. In the human papillomavirus (HPV) subtype 16 E7 vaccine model, an intranasal, but not an intramuscular vaccine, promotes specific infiltration of CD103^+^CD49a^+^CD8^+^ T cells in broncho-alveolar lavage and also in an HPV16-E7-expressing tongue tumor [36]. The advantage of site-specific vaccination compared to systemic immunization for inducing local immunity and T_RM_ cells could be explained by imprinting of T cells induced after initial activation by tissue APC. Indeed, specific DC have been shown to be involved in upregulation of specific molecular homing programs on T cells. Along the same lines, lung but not splenic DC were able to drive CD49a expression in vitro after intranasal vaccination of OT-I mice [36]. At present, DC seem to provide differentiation and homing signals to the initial site of priming through production of specific cytokines. This is supported by the observation that local DC induce α_4_β_7_ on CD8^+^ T cells through their secretion of retinoic acid after a cervico-vaginal boost [67]. Similarly, respiratory CD103^+^ DC promote CD103 upregulation upon CD8^+^ T-cell activation in a TGF-β-dependent manner [75]. In a virus model, Iborra et al. also demonstrated that DNGR-1^+^ (Clec9a) DC provide essential cytokine signals for the development of a T_RM_ phenotype [76]. Overall, the molecular mechanisms involved in T_RM_ priming are highly complex due to the wide diversity of experimental models, tissues, cells and markers studied with no standardization. It appears that DC and the tissue microenvironment are both implicated in the induction of a particular T_RM_ phenotype. Future studies should define the coordinated role of the various parameters (DC, cytokines, stroma signals and sequences of the various steps) in generating T_RM_. Such insights may help to better understand how to prime these memory T cells.

## Role of T_RM_ in immune surveillance and immunotherapy

### T_RM_ cells can be located in solid tumors

Mueller and Mackay [77] revealed that T_RM_ cells are mainly present in non-lymphoid tissues and express CD69 and the CD103 integrin. These cells are also found in various tumors, including melanoma [78], lung cancer [27, 39], urothelial cell carcinoma [29] and endometrial adenocarcinoma [79]. Tumors with a high density of CD8^+^ T cells showed enrichment for transcripts linked to tissue-T-cell-residency, such as CD103 [38]. However, there exists phenotypic heterogeneity in T_RM_ cells according to their location and tumor histological subtype. In all subtypes of endometrial adenocarcinoma, CD8^+^ TIL were present in both the tumor epithelium and stromal areas, but the frequency of CD8^+^CD103^+^ T cells was significantly higher in the tumor epithelium than in the stroma [27, 29, 79]. Most CD103^+^ cells in the tumor microenvironment co-express the CD8 molecule, whereas CD8^+^ TIL located in the stroma were mainly negative for CD103 integrin. Nizard et al. found that 70% of intra-tumoral CD8^+^ T cells expressed CD103, whereas the integrin is found on only 41% of stromal CD8^+^ T cells [39]. Conversely, CD103^+^ cells in the healthy endometrium were negative for CD3 and CD16, suggesting a non-T-cell origin [29].

CD103 binds to E-cadherin expressed on the surface of epithelial cells [52]; this binding may be involved in retention of these cells in the epithelial tissue, as well as in solid tumors [80]. Interestingly, in some studies, distribution of CD103^+^ TIL was positively associated with E-cadherin expression on tumor cells [29]. However, in other studies, there was no obvious correlation between E-cadherin staining intensity and the presence of CD103^+^ TIL, suggesting that other factors are also determinant in their infiltration [26, 81].

### T_RM_ cells control tumor growth

In a preclinical model of spontaneous breast cancer, a natural immune response involving resident innate lymphoid cells (ILC) close to ILC1 and TCR-positive cells was described [82]. These cells do not recirculate and delay tumor growth. Tumor growth control can be explained by the fact that CD49a^+^ and CD103^+^ cells are highly activated and exhibit more satisfactory effector functions than conventional CD8^+^ T cells [27, 38, 83]. These resident cells produce more IFN-γ and granzyme B than their integrin-negative counterparts. Accordingly, significantly impaired tumor control was observed in mice treated with either anti-CD49a or anti-CD103 antibodies [36, 83].

Following vaccination against orthotopic tumors, it has been shown that T_RM_ cells are required for the efficacy of a cancer vaccine. Indeed, in a preclinical head and neck cancer model expressing E6-E7 proteins from HPV, the mucosal (intranasal) delivery of a vaccine (the B subunit of Shiga toxin coupled with the E7 protein from HPV16) was efficient at eliciting local T_RM_ cells and control of tumor growth [36]. Interestingly, a body of experiments demonstrated the role of T_RM_ cells in the efficacy of this cancer vaccine. Indeed, depletion of CD49a^+^ T_RM_ cells with an antibody hampered infiltration of T_RM_ in mucosal tumors and partially inhibited the efficacy of intranasal vaccination to control mucosal tongue tumors. Similar results were obtained by Murray et al., using anti-CD49a mAb in a melanoma model [83]. In one of our group's study, the co-administration of an anti-TGF-β antibody with the vaccine reduced the number of T_RM_ cells and control of tumor growth by the vaccine [39]. By blocking recruitment of effector T cells arising from the lymphoid organs with the FTY720 drug, which downmodulates the S1PR1 molecule, it has been demonstrated that T_RM_ cells induced by intranasal vaccination are able to control tumors [39]. Lastly, in a parabiosis mouse model, one of our groups showed that T_RM_ cells induced by intranasal vaccination are required to control orthotopic head and neck tumor growth [39]. These results obtained with parabiosis experiments have been reproduced by the group of Sancho [10]. Together, these observations suggest that the absence of local T_RM_ induction correlates with lower vaccine efficiency; they highlight the crucial role of these cells in tumor control. In line with these results, it was reported that cervico-vaginal boost with an HPV vaccine after a systemic (intramuscular) prime was more efficient at eliciting local cervical T_RM_ cells, which was correlated with better mouse survival than that observed with an intramuscular boost [67]. In another model, it was shown that the number of T_RM_ cells in tissues gradually increased after each boost [20], underlining the need for repeated injections.

At the present time, anti-tumor vaccine protocols almost exclusively use systemic administration with no significant clinical results, whereas various cancers are located in mucosal sites (lung, head and neck and urogenital). Therefore, it is important to reevaluate the advantage of local delivery by better understanding T_RM_ cell physiology [84]. It should be mentioned that other non-T_RM_ effector cells might also play a role in control of mucosal tumors [85], and the presence of T_RM_ is not always sufficient to cure high grade cervical dysplasia after vaccination, likely due to the presence of immunosuppressive mechanisms or their insufficient local number [86].

### Prognostic value of T_RM_ cells

The CD8^+^ T_RM_ subset has emerged as a predictive marker of survival in several human epithelial cancers [26, 27, 29, 38, 87]. In this regard, one of our groups first demonstrated that an enhanced CD103^+^ TIL subset correlates with improved early-stage non-small-cell lung carcinoma (NSCLC) patient survival [27]. The predictive value of T_RM_ was also demonstrated in ovarian, breast and bladder cancers [26, 28, 64, 88]. Indeed, in a large cohort of high-grade serous ovarian cancers, CD103^+^ TIL were associated with improved patient survival [26]. Moreover, the expression of CD103 on TIL was associated with improved overall and recurrence-free survival in a retrospective cohort of urothelial cell carcinoma patients [29]. This integrin also appeared to be a biomarker of favorable prognosis in a large cohort of breast cancer patients [89]. However, the CD103 biomarker could also be expressed by CD4^+^ T cells and DC, which introduces bias in interpretation of results without double immunostaining with anti-CD8 mAb. The epithelial location of CD103^+^ TIL is an even more significant prognosis marker compared to the stromal location, suggesting that intraepithelial CD8^+^CD103^+^ cells encompass a higher proportion of tumor-specific T_RM_ cells [27, 89]. This intratumoral infiltration of CD103^+^ TIL was associated with expression of E-cadherin on tumor cells in bladder cancer [29], but not in ovarian or breast cancer [26, 89].

Since it is well known that CD8^+^ T-cell infiltration is associated with better clinical outcome in many cancers [90], comparative analysis of the prognostic value of T_RM_ and CD8^+^ T cells has been lacking. Two recent studies, including one from one of our groups, demonstrated that, in two independent cohorts of lung cancer patients, T_RM_ cells were correlated with patient survival in both univariate and multivariate analysis, and this effect was independent of CD8^+^ T cells [38, 39].

### T_RM_ in adoptive cell transfer therapy

With respect to adoptive cell transfer, Milner et al., identified the transcription factor Runx3 as critical for the establishment of T_RM_ cell populations in various normal tissues and in cancer [15]. In a preclinical model of melanoma, adoptive transfer of CD8^+^ TIL lacking expression of Runx3 and which did not exhibit a T_RM_ cell phenotype resulted in uncontrolled tumor growth and low animal survival. In contrast, when anti-tumor CD8^+^ T cells overexpressing Runx3 were transferred in vivo, tumor growth was inhibited, and mouse survival improved [15]. Thus, adoptive cell therapy with anti-tumor CD8^+^ tumor-infiltrating T lymphocytes displaying a T_RM_ phenotype improves the efficacy of this immunotherapy approach. Although T_RM_ cells lacking the expression of CD103 integrin have been observed, the transfer of CD103-deficient T cells has also been used to demonstrate the role of T_RM_ cells in tumor progression control. In this setting, it has been shown that T_RM_ cells are required for animal protection [78].

### T_RM_ cells are potential effectors of checkpoint blockade immunotherapy

One of our groups was the first to report preferential expression of immune checkpoint receptors (PD-1 and TIM-3) and costimulatory molecules (ICOS) in T_RM_ cells from lung cancer patients [27], extending similar results observed in T_RM_ from normal tissues [37, 91]. These results have been confirmed in other cancers, both in mice and in humans [27, 38, 39, 83, 92]. In human cervical cancer, a strong correlation between expression of CD103 and exhaustion molecules such as PD-1, TIGIT, LAG-3 and Tim-3 has been observed using the Cancer Genome Atlas [93]. This result has been confirmed at the protein level in ovarian and endometrial adenocarcinomas [26, 79]. Other checkpoint receptors (NKG2A, CD39, adenosine receptor A2AR and SPRY1) may also be preferentially expressed by T_RM_ cells [14, 38]. Remarkably, one of our groups showed that blockade of PD-1 on T_RM_ cells freshly isolated from human lung carcinomas strongly promotes cytolytic activity toward autologous tumor cells ex vivo [27]. Moreover, anti-MHC-I and anti-CD103 neutralizing antibodies dramatically inhibited target cell killing by autologous TIL pretreated with anti-PD-1, further emphasizing that CD8^+^CD103^+^ T_RM_ cells were exhausted tumor-specific T lymphocytes, which could be rescued by blocking PD-1 signals resulting in T-cell activation and autologous tumor cell killing [27]. In line with these results, after infection in mucosal tissues, T_RM_ cells can proliferate and generate a second pool of T_RM_, strongly suggesting that they have the ability to be activated in situ for better control of local danger [94]. Therefore, their exhausted phenotype does not preclude their sensitivity to reactivation and invigoration [95]. Consistently, recent results revealed expansion of T_RM_ cells in melanoma patients responding to anti-PD-1 immunotherapy [96].

Taken together, their expression of checkpoint receptors, their strategic location in close tumor contact and their ability to proliferate in situ after a local stimulus suggest that T_RM_ cells are enriched in tumor-specific CD8^+^ T cells, making them possible effectors of anti-PD-1/anti-PD-L1 cancer immunotherapy.

## Conclusion

Overall, T_RM_ cells appear to represent important components in cancer immunology. Their presence in the tumor microenvironment is correlated with good clinical outcome and may identify spontaneously immunogenic tumors. Moreover, their induction by cancer vaccines or other immunotherapeutic approaches may be critical for the success of immunotherapy. Several arguments strongly suggest that they may be the target of anti-PD-1/PD-L1 mAb therapies in various human cancers.
